# Lipoaspirate fluid derived factors and extracellular vesicles accelerate wound healing in a rat burn model

**DOI:** 10.3389/fbioe.2023.1185251

**Published:** 2023-06-22

**Authors:** Yue Wu, Pengyu Hong, Pan Liu, Qi Zhang, Yue Zhang, Baohua Yang, Huixing Liu, Lei Liu, Weidong Tian, Mei Yu

**Affiliations:** ^1^ State Key Laboratory of Oral Disease, National Engineering Laboratory for Oral Regenerative Medicine, National Clinical Research Center for Oral Diseases, West China School of Stomatology, Sichuan University, Chengdu, China; ^2^ Engineering Research Center of Oral Translational Medicine, Ministry of Education, Sichuan University, Chengdu, China; ^3^ Department of Oral and Maxillofacial Surgery, West China Hospital of Stomatology, Sichuan University, Chengdu, China; ^4^ Sichuan Huamel Zixin Medical Aesthetic Hospital, Chengdu, Sichuan, China

**Keywords:** extracellular vesicles, adipokines, lipoaspirate fluid, rat burn model, wound healing

## Abstract

**Background:** The regenerative capabilities of derivatives derived from the fat layer of lipoaspirate have been demonstrated. However, the large volume of lipoaspirate fluid has not attracted extensive attention in clinical applications. In this study, we aimed to isolate the factors and extracellular vesicles from human lipoaspirate fluid and evaluate their potential therapeutic efficacy.

**Methods:** Lipoaspirate fluid derived factors and extracellular vesicles (LF-FVs) were prepared from human lipoaspirate and characterized by nanoparticle tracking analysis, size-exclusion chromatography and adipokine antibody arrays. The therapeutic potential of LF-FVs was evaluated on fibroblasts *in vitro* and rat burn model *in vivo*. Wound healing process was recorded on days 2, 4, 8, 10, 12 and 16 post-treatment. The scar formation was analyzed by histology, immunofluorescent staining and scar-related gene expression at day 35 post-treatment.

**Results:** The results of nanoparticle tracking analysis and size-exclusion chromatography indicated that LF-FVs were enriched with proteins and extracellular vesicles. Specific adipokines (adiponectin and IGF-1) were detected in LF-FVs. *In vitro*, LF-FVs augmented the proliferation and migration of fibroblasts in a dose-dependent manner. *In vivo*, the results showed that LF-FVs significantly accelerated burn wound healing. Moreover, LF-FVs improved the quality of wound healing, including regenerating cutaneous appendages (hair follicles and sebaceous glands) and decreasing scar formation in the healed skin.

**Conclusion:** LF-FVs were successfully prepared from lipoaspirate liquid, which were cell-free and enriched with extracellular vesicles. Additionally, they were found to improve wound healing in a rat burn model, suggesting that LF-FVs could be potentially used for wound regeneration in clinical settings.

## 1 Introduction

Burn injuries represent a significant disease burden on the world’s population, with nearly 9 million injuries and an estimated 120,000–180,000 deaths annually (James et al., 2020). At present, standard nursing, early excision, and skin grafting have been used to treat burn injuries (Las Heras et al., 2020; Shahin et al., 2020), however, the long-term medical treatment (Bailey et al., 2019) and severe complications such as permanent scarring and disfigurement (Tan et al., 2017) cause heavy burden to patients, in addition, skin grafting has the disadvantages such as limited source, additional surgical trauma and possible complications including necrosis, contraction and infection of the donor area (Stekelenburg et al., 2016; Ozhathil et al., 2021). Therefore, there is an increasing need for a more effective and ideal treatment strategy.

Adipose tissue has garnered special attention in wound healing and scar repair as a rich source of bioactive substances (Cai et al., 2020; Guo et al., 2022). Scholars have found that fat grafting could significantly accelerate burn wound healing and improve scar quality (Klinger et al., 2008; Klinger et al., 2020). He et al. confirmed the inductive effect of cell-free adipose tissue extract prepared from human subcutaneous adipose tissue on wound healing in C57BL/6 mice (He et al., 2019). Wang et al. obtained adipose tissue extract from healthy female adipose tissue and demonstrated that it could accelerate diabetic wound healing in db/db mice through pro-angiogenic and anti-inflammatory activities (Wang et al., 2020). Paracrine cytokines and extracellular vesicles (EVs) were reportedly the main factors through which adipose tissue and its derivatives exert their biological effects (Bellei et al., 2022; Li et al., 2022). Lipoaspirate waste is typically divided into two parts: a fat layer and a liquid layer. Various adipose tissue derivatives (adipose-derived stem cells conditioned medium, adipose-derived stem cells extracellular vesicles and adipose tissue extract, *etc.*) are produced from the fat layer of lipoaspirate waste, which play an important role in the field of tissue repair and regenerative medicine, particularly in scar repair and wound healing (Cai et al., 2020; Bellei et al., 2022). Despite the efficient therapeutic efficacy exhibited by the secretome of these cultured cells/tissue, several hurdles must be addressed before translating this therapy from bench-to-bedside. For example, isolation of cells using enzymatic digestion that increases the risk of biological contamination and the secretome harvested from cultured cells/tissues requires considerable cell expansion, specific laboratory equipment and time-consuming steps (Mazini et al., 2020; Poulos, 2018). Based on the effectiveness and feasibility of adipose-derived therapies in the field of wound healing and scar prevention, we speculated that a variety of bioactive factors, including growth factors and extracellular vesicles, could be extracted from the discarded lipoaspirate fluids and play a therapeutic role in repairing burn skin wounds.

This study presented a novel method to obtain a cell-free liquid portion from lipoaspirate fluids, the “lipoaspirate fluid derived factors and extracellular vesicles” (LF-FVs). The contents of LF-FVs were detected and the therapeutic effects of LF-FVs on burn wound healing and scar reduction in rats were investigated with two administration methods (local injection and topical spray).

## 2 Materials and methods

### 2.1 LF-FVs preparation

Healthy donors (1) no infectious diseases such as syphilis and acquired immune deficiency syndrome (AIDS) (2) 18<BMI<30 were included in this study. Human lipoaspirate fluid was collected from 4 patients (4 women) as waste material from liposuction surgery in Sichuan HUAMEI ZIXIN medical aesthetic hospital after written consent. The mean age was 31 years (range, 22–40 years). Detailed information about patients was listed in Supplementary Table S1. The procedures were approved by the Institutional Review Board of Sichuan University West China Hospital of Stomatology (Approval number, WCHSIRB-D-2021-028).

**FIGURE 1:**
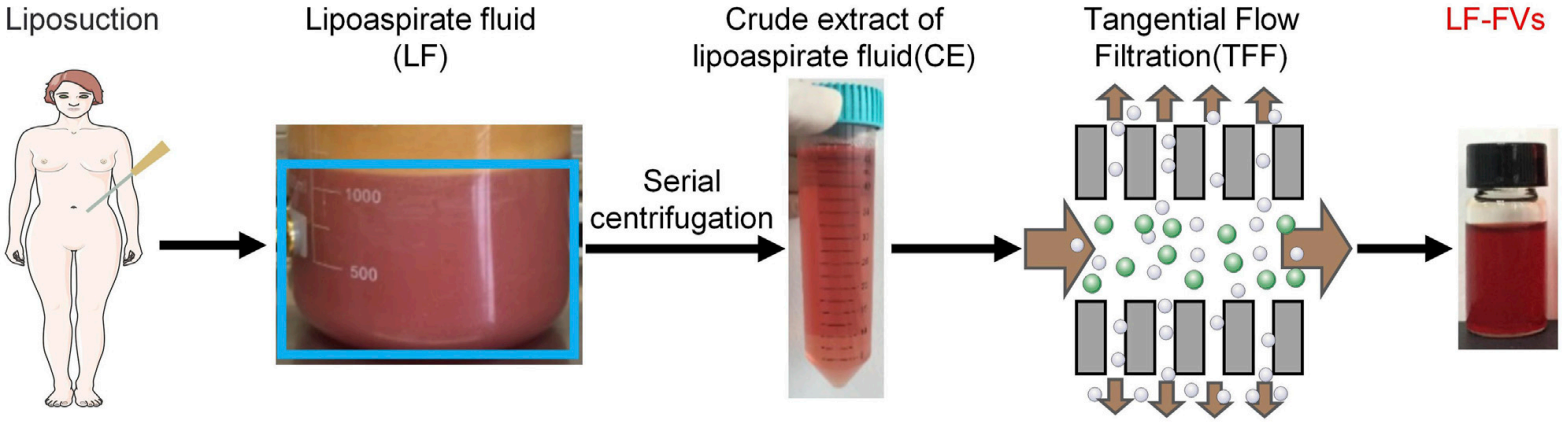
Schematic illustration of LF-FVs preparation.

After collection, the lipoaspirate fluid is differentially centrifuged at 300 g for 10 min and 2,000 g for 10 min to remove cells and tissue debris. The supernatant is then further centrifuged at 15,000 g for 1 h to remove the fraction of large EVs. After the third centrifugation, the supernatant was collected to obtain crude extract of lipoaspirate fluid (CE). The volume of CE was further concentrated through tangential flow filtration (TFF) using 500 kDa molecular weight cut-off capsule (Millipore), the remaining concentrated fluid was named as LF-FVs (Figure 1).

### 2.2 Nanoparticle tracking analysis (NTA)

The particle concentration and size distribution of isolated LF-FVs samples were determined with Zeta View (Particle Metrix, Germany) as per the manufacturer’s instructions.

### 2.3 Size-exclusion chromatography (SEC)

1 mL LF-FVs were overlaid on top of the Sepharose-4B (4B200-100mL, CAS:9012-36-6) in 10 mL disposable plastic column (Thermo, 29924). A total of 30 sequential fractions of 500 μL were collected according to the manufacturer’s protocol. The particle number and protein concentration of SEC fractions were detected by NTA, and the bicinchoninic protein assay (BCA Protein Assay Kit, KeyGEN BioTECH) respectively.

### 2.4 Adipokine antibody array

Adipokines in LF-FVs were analyzed using Quantibody^®^ Human Obesity Array 3 (QAH-ADI-3) (RayBiotech, United States) according to the manufacturer’s protocol. The fluorescence signals were visualized through a laser scanner equipped with a Cy3 wavelength. Signal intensities were quantified with the microarray analysis software (GenePix).

### 2.5 Cell culture

Fibroblasts are critical in supporting wound healing. One of the most available sources of human fibroblasts is the foreskin (Bainbridge, 2013; Ramenzoni et al., 2017). In the current study, human foreskin fibroblasts (HFF) were purchased from Shanghai Institute of Biochemistry and Cell Biology (China) and cultured in Dulbecco’s modified Eagle’s medium supplemented with 10% fetal bovine serum and penicillin/streptomycin (P/S; 1000 UI/mL) at 37°C in humidified air containing 5% CO_2_.

### 2.6 Cell proliferation assay

1 x 10^3^ HFF were seeded into 96-well plates and cultured with medium containing LF-FVs at the appropriate concentrations (0, 10, 50, 100, 250 μg/mL) (0.1 mL per well) before Cell Counting Kit-8 assay (CCK8, KeyGEN BioTECH, Nanjing, China). Untreated cells (0 μg/mL) served as the control group. The optical density (OD) value was detected at 450 nm using the Multiskan Go Spectrophotometer (Thermo Fisher Scientific) from day 1 to day 6 (*n* = 5).

### 2.7 Cell scratch assay

Confluent HFF monolayers were scratched with a sterile 200 μL pipette tip, washed twice in PBS to remove debris and cocultured with LF-FVs at the indicated concentrations (0, 10, 50, 100, 250 μg/mL). The initial (0 h after scratching) and final (16 h after scratching) images were captured by phase-contrast microscope (Olympus), and the scratch area was analyzed by ImageJ software. The data were expressed as the relative percentage of wound healing = [(A_0_ - A_t_)/A_0_] × 100%, where A_0_ is the initial wound area (t = 0) and A_t_ is remaining wound area at 16 h.

### 2.8 Transwell assay

Transwell assay was performed using 24-well transwell inserts (8 μm pore, Corning, Unites States). 2 x 10^3^ HFF were plated into the upper chamber, then cultured in 500 μL complete medium (containing 10% FBS) supplementing LF-FVs at the indicated concentrations (0, 10, 50, 100, 250 μg/mL). After incubation for 24 h, non-migrated cells were removed by cotton swab and migrated cells were fixed with 4% paraformaldehyde and stained with 0.1% crystal violet (Sigma, United States). The number of migrated cells was calculated under an optical microscope at a ×100 magnification (Olympus).

### 2.9 *In vivo* experiments

The rats were purchased from Dashuo experimental animal Co., Ltd. (Chengdu, China). All operations of animals were approved by the Institutional Review Board of Sichuan University West China Hospital of Stomatology (WCHSIRB-D-2020-269). The third-degree burns were performed on the dorsum of SD (Sprague Dawley) male rats as previously described (Foubert et al., 2016; Dong et al., 2020; Zhou et al., 2019). Briefly, male 5-week-old SD rats (*n* = 27) were anesthetized by intraperitoneal injection of 1% pentobarbital. One burn (20 mm in diameter) was made on the shaved dorsum of each rat using a round metal hot iron (diameter, 20 mm) of a temperature-controlled burner (YLS-5Q, China). Two days after burning, wound sites were excised to create a fresh full-thickness wound. 27 rats were randomly divided into 3 groups: (1) control group (PBS injected subdermally into the superficial fascia of the wound, 0.25 mL/wound, *n* = 9), (2) local injection of LF-FVs (5 mg LF-FVs/0.25 mL/wound, *n* = 9) and (3) topical spray of LF-FVs directly onto the wound bed (5 mg LF-FVs/0.25 mL/wound, *n* = 9). The dosage of LF-FVs for local injection and topical spray was based on the preliminary results of the pre-experiment *in vivo*. For local injection, LF-FVs were locally injected subdermally into the superficial fascia around the wounds at 5 injection sites (50 μL per site) to provide a dose of 5 mg LF-FVs. For topical spray, 250 μL LF-FVs were sprayed directly onto the wound bed, after each spray, the rats were positioned in hand until sprayed solution dried. Local injection of LF-FVs were administered every 2 days, and the topical spray of LF-FVs were administered every day. After the treatments, the wounds were exposed while the rats were kept individually with food and water in animal room. The wound areas were photographed on days 2, 4, 8, 10, 12, 16 and 35 post-treatment and quantified using ImageJ software. The wound area at different time points (Wound Area_T_) was compared to the wound area on the excision day (Wound Area_0_) and indicated as (% of wound = Wound Area_T_/Wound Area_0_ x 100%). Additionally, the area of unhealed wounds at different time points was represented by a contour-like wound tracing pattern to represent the speed of burn wound healing more clearly.

The skin-wound repair process was classically divided into four phases, namely, hemostasis (hours), inflammation (days), proliferation (1–2 weeks), and remodeling (>2 weeks). We assumed that 35 days after wounding could be considered in the progress of the remodeling phase (Hu et al., 2021). At 35 days, the rats were euthanized via cervical dislocation under general anesthesia and skin tissues were harvested for further testing. For each group, 4 of 9 skin samples were preserved in a 4% paraformaldehyde for histology; 4 skin samples were dissolved in Buffer RL1 (Vazyme, China) for real-time PCR; and 1 sample was dissolved in a RIPA Lysis Buffer (KeyGEN, China) for western blot.

### 2.10 Quantitative real-time polymerase chain reaction (qRT-PCR)

RNA was isolated from skin tissue using FastPure^®^ Cell/Tissue Total RNA Isolation Kit (Vazyme, China) following the manufacturer’s protocol. qRT-PCR was performed with the SYBR Green PCR master mix (Vazyme, China) using the QuantStudio™ 6 Flex Real-Time PCR System (Applied Biosystems). Relative gene expressions were analyzed using the 2^−ΔΔCT^ method with GAPDH as the endogenous control (Supplementary Table S2).

### 2.11 Western blot analysis

Briefly, the skin tissue samples were dissolved in RIPA Lysis Buffer (KeyGEN, China), resolved on a 10% polyacrylamide gel, and blotted on to a nitrocellulose membrane. The membranes were blocked and then incubated with primary antibodies against TGF-β1 (Abcam, ab92486), α-SMA (ab5694), COL I (ab270993) and β-actin (Zen Bioscience, 200068-8F10) at 4°C overnight, followed by horseradish peroxidase (HRP)-conjugated secondary antibodies for 1 h at room temperature. Immobilon Western Chemiluminescent HRP Substrate (Millipore) was used for the detection following the manufacturer’s instructions. β-actin was used as an internal loading control. Signals were visualized with an ImageQuant LAS 4000 mini (GE Healthcare).

### 2.12 Histological staining

Wound skin samples at 35 days were fixed overnight with 4% paraformaldehyde in PBS at 4°C, dehydrated using graded ethanol and paraffin-embedded and sectioned into 5 µm slides for H&E staining, masson’s trichrome staining and picrosirius red staining (Chantre et al., 2018; Wang et al., 2017). To assess the relative degree of scar formation, the epidermal thickness was quantified according to H&E staining under an Olympus VS.200 Whole Slide Scanner. The thickness of the epidermis is defined as the distance between the stratum basal and stratum granulosum and measured manually using OlyVIA software based on the scale bar. To assess the relative degree of skin regeneration, skin appendage (hair follicles and sebaceous glands) density was determined according to masson’s trichrome staining. Five fields of each tissue section containing the epidermis in the healed skin were randomly selected, then the hair follicles and sebaceous glands were counted per field 500 μm × 1000 μm with OlyVIA software. The mean and standard error was showed from 4 skin samples. Following picrosirius red staining, the images of the stained sections were obtained by Olympus VS. 200 Whole Slide Scanner. All counts and measurements were finished by two authors in a double-blinded fashion.

### 2.13 Immunofluorescence staining

The disappearance of myofibroblasts is important for the prevention of scar formation, so the presence of myofibroblasts in wound tissue was detected by immunofluorescence staining. Briefly, the sections were incubated with primary antibodies anti-α-SMA (Abcam, ab5694) and anti-COL I (Abcam, ab270993) at 4°C overnight. After PBST washing, sections were incubated with secondary antibody Alexa Fluor 555 goat anti-rabbit (Invitrogen, A21428) and Alexa Fluor 488 goat anti-rabbit (Invitrogen, A11008) at room temperature for 1 h. The images of the stained sections were obtained by confocal microscopy at ×200 magnification (Olympus FV1000, Japan).

### 2.14 Statistics

Statistical analyses were performed using GraphPad Prism software (v.6.0, GraphPad). Results are expressed as mean value ± standard deviation. One-way ANOVA with Tukey posthoc test was used to determine the level of significance. In all figures, we used ∗ to denote *p*-values, Values of *p* < 0.05 were accepted as significant and no significance *p* > 0.05.

## 3 Results

### 3.1 Characteristics of LF-FVs

**FIGURE 2:**
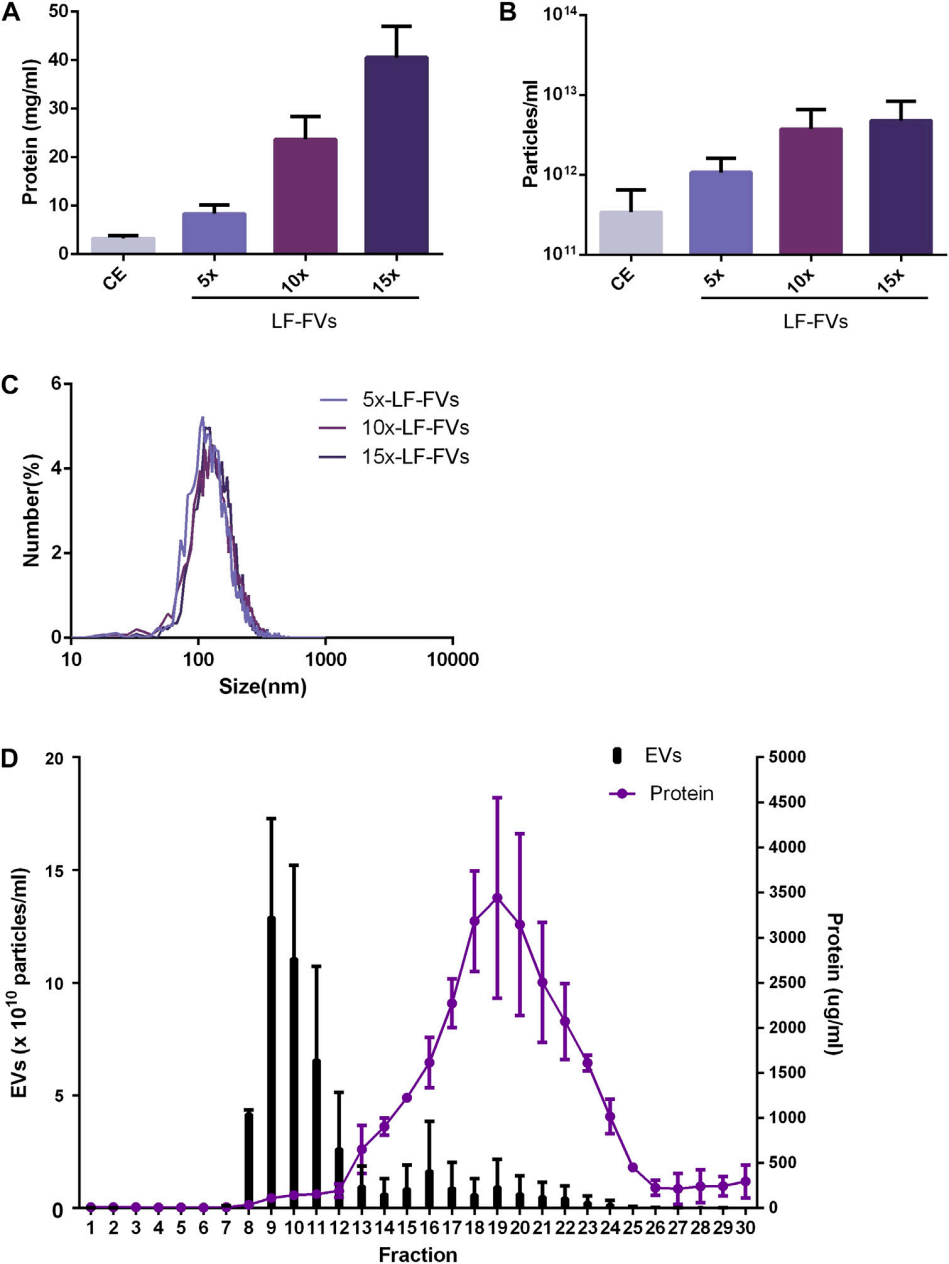
Characterization of LF-FVs with indicated TFF concentration fold. **(A)** Protein concentration was determined by the bicinchoninic protein assay. **(B)** The concentration of particles was detected by NTA (particles/mL). **(C)** The size distribution of 5x, 10x and 15x LF-FVs was measured by NTA. **(D)** particle and protein concentration measurements of size-exclusion chromatography fractions from LF-FVs.

To evaluate the optimal TFF parameter, the volumes of crude extract from 4 patients were concentrated at 5 fold, 10 fold or 15 fold through TFF to obtain 5x, 10x or 15x LF-FVs. The protein concentration in CE, 5x, 10x and 15x LF-FVs were 3.29 ± 0.53, 8.35 ± 1.79, 23.70 ± 4.75, 40.57 ± 6.44 mg/mL respectively (Figure 2A). The particle numbers in CE, 5x, 10x and 15x LF-FVs were 3.43 ± 3.04, 10.72 ± 5.39, 51.75 ± 17.91 and 60.67 ± 29.94 (x10^11^ particles/mL), respectively (Figure 2B). The median diameter of the particles in 5x, 10x and 15x LF-FVs was 131.00 ± 4.00, 138.00 ± 4.41 and 138.40 ± 7.51 nm respectively (Figure 2C).

10x LF-FVs from 4 patients were further analyzed by size-exclusion chromatography respectively. The protein concentration increased in fractions 12-25, with a peak at fraction 19. Fractions 8-12 were accompanied by the increase in particle concentration, with a peak at fraction 9. The results suggested that both soluble proteins and EVs were enriched in LF-FVs (Figure 2D). To further quantify the soluble proteins in LF-FVs, Quantibody^®^ Human Obesity Array 3 were applied for 10x LF-FVs from 4 patients respectively. Among the 40 specific adipokines, two adipokines (adiponectin and IGF-1) were highly expressed in LF-FVs, of which concentrations were 4.60 ± 0.10 and 1.46 ± 0.33 (x10^5^ pg/mL) respectively (Table 1). However, a few adipokines (such as IL-8, IL-1b, AgRP and Pepsinogen II) were not detected.

**TABLE 1:** | The concentrations of adipokines in LF-FVs (pg/ml).

Adipokines	LF-FVs-1	LF-FVs-2	LF-FVs-3	LF-FVs-4	Mean
Adiponectin	456,645.40	466,811.00	447,634.50	468,411.40	459875.58
TSP-1	156,188.40	123,340.70	94,174.70	201,408.40	143778.05
IGF-1	159,129.80	161,233.10	96,734.30	166,776.00	145968.30
Adipsin	70,722.60	72,858.70	59,598.60	74,250.80	69357.67
Chemerin	15,975.00	42,234.20	45,800.20	35,486.70	34874.03
CRP	13,276.80	8,560.70	10,688.90	44,579.60	19276.50
SAA	13,160.90	6,026.20	5,164.60	37,527.80	15469.88
Leptin	3,133.30	158.60	2,927.30	12,668.20	4721.85
RBP4	9,459.20	10,230.20	9,214.00	10,069.10	9743.13
Procalcitonin	6,563.90	11,310.60	8,944.60	9,197.10	9004.05
ANGPTL4	5,702.00	1,724.30	4,498.90	5,865.40	4447.65
TGFb1	2,843.20	2,354.60	3,560.00	2,753.50	2877.83
Pepsinogen I	1,276.90	1,683.60	852.30	1,018.80	1207.90
Insulin	1,651.20	1,373.20	1,205.00	1,254.70	1371.03
MSP	1,486.40	837.40	91.70	1,528.50	986.00
PAI-1	495.40	575.80	78.40	1,212.10	590.43
IGFBP-2	1,050.30	1,439.40	913.50	1,154.00	1139.30
TNF RI	472.40	989.30	237.40	549.00	562.03
Lipocalin-2	579.90	431.40	455.80	934.60	600.43
IL-1ra	540.50	546.60	413.00	310.80	452.73
IGFBP-1	188.60	455.10	482.20	467.70	398.40
GH	471.00	489.20	418.90	354.30	433.35
TNF RII	332.90	434.40	111.90	342.00	305.30
OPG	310.90	371.30	3.30	118.00	200.88
IFNg	365.30	242.60	322.90	282.10	303.23
IL-6	343.90	322.90	350.80	310.00	331.90
TNFa	231.60	222.10	176.80	189.50	205.00
Resistin	54.90	52.70	89.60	63.90	65.28
IL-10	65.50	44.60	51.90	62.30	56.08
VEGF	16.80	26.60	38.50	51.00	33.23
Pepsinogen II	14.10	0.00	138.30	0.00	38.10
IL-12p70	25.50	16.90	13.40	15.40	17.80
BDNF	15.40	20.10	13.50	21.70	17.68
IL-12p40	11.70	19.10	12.80	10.50	13.53
AgRP	6.30	9.50	5.50	0.00	5.33
PDGF-BB	3.50	2.40	2.90	6.50	3.83
Prolactin	7.20	0.00	749.20	9.30	191.43
RANTES	1.40	0.00	0.40	1.90	0.93
IL-1b	1.40	1.20	1.50	0.00	1.03
IL-8	0.00	0.00	0.00	0.00	0.00

To avoid the inconsistent results caused by heterogeneity of LF-FVs from different patients, the 10x LF-FVs from 4 patients were pooled for subsequent analysis.

### 3.2 LF-FVs promoted HFF proliferation and migration *in vitro*

**FIGURE 3:**
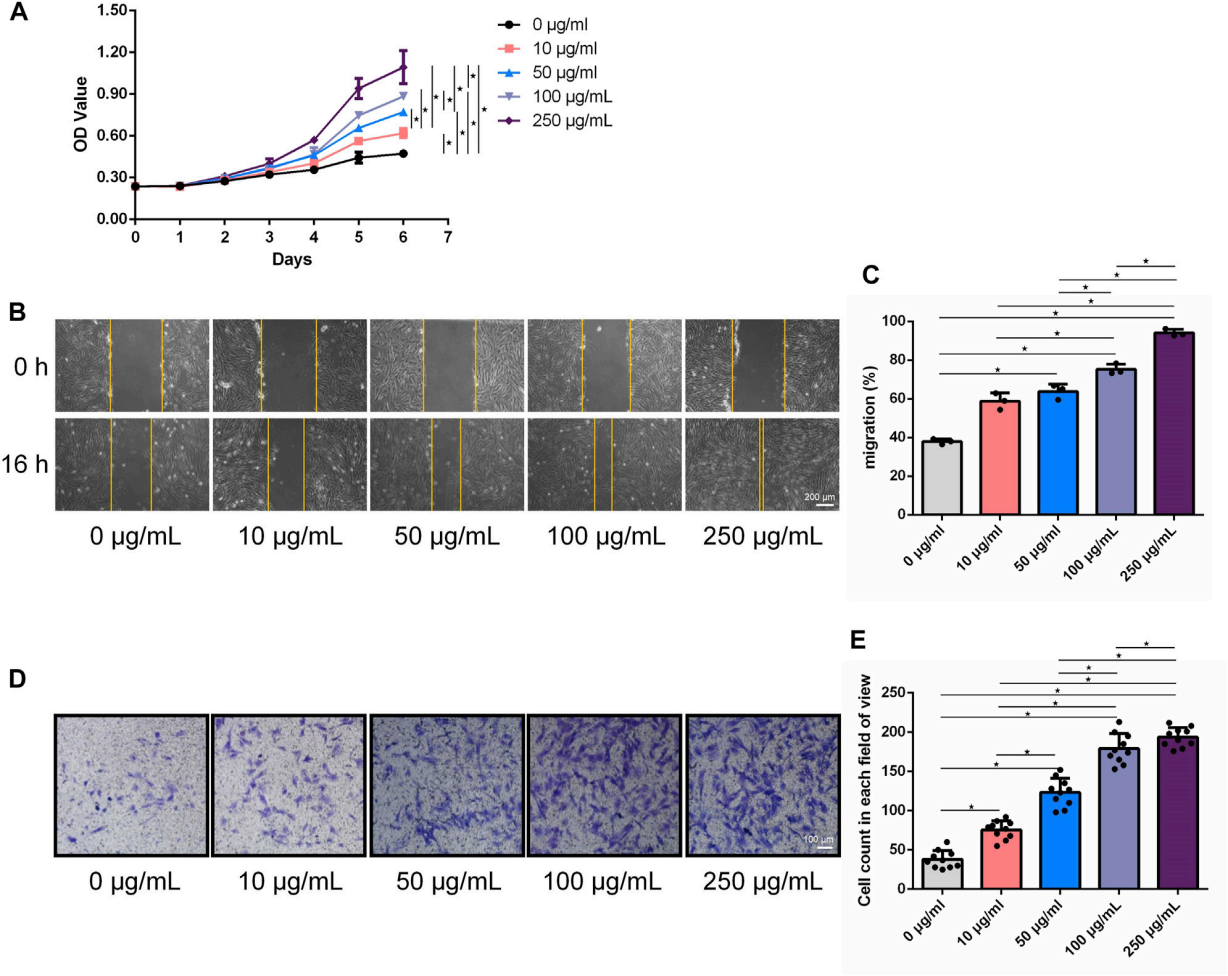
LF-FVs promoted HFF proliferation and migration. **(A)** Growth curves of HFF which were co-cultured with LF-FVs at indicated concentrations. **(B)** Representative images of scratch assay in HFF that co-cultured with LF-FVs at indicated concentrations and **(C)** relative quantification. Scale bars = 200 µm. **(D)** The migration of HFF that co-cultured with LF-FVs at indicated concentrations was detected by the transwell assay and **(E)** relative quantification. Scale bars = 100 µm. (^∗^ *p* < 0.05).

Growth curves of HFF cultured with LF-FVs at different concentrations were plotted with CCK-8 assay (Figure 3A). At day 6, the proliferation of HFF was promoted by 31.35% ± 12.74%, 63.16% ± 8.29%, 87.49% ± 14.65% and 130.55% ± 16.86% with the addition of LF-FVs at 10, 50, 100 and 250 μg/mL, respectively (*p* < 0.05). In the scratch assay, the relative migrated HFF cells coverage at 16 h was 37.91% ± 1.38%. With the addition of LF-FVs at 10, 50, 100 and 250 μg/mL, cells coverage was 58.75% ± 4.27%, 63.76% ± 3.81%, 75.18% ± 2.74%, 94.04% ± 1.83% respectively (*p* < 0.05) (Figures 3B; C). In transwell assay, the numbers of migrated cells were 38.00 ± 11.24, 75.60 ± 11.55, 123.30 ± 17.94, 179.00 ± 19.18 and 193.70 ± 12.28 in the group treated with 0, 10, 50, 100, 250 μg/mL LF-FVs respectively (Figures 3D, E).

### 3.3 LF-FVs accelerated wound healing in burn wound

**FIGURE 4:**
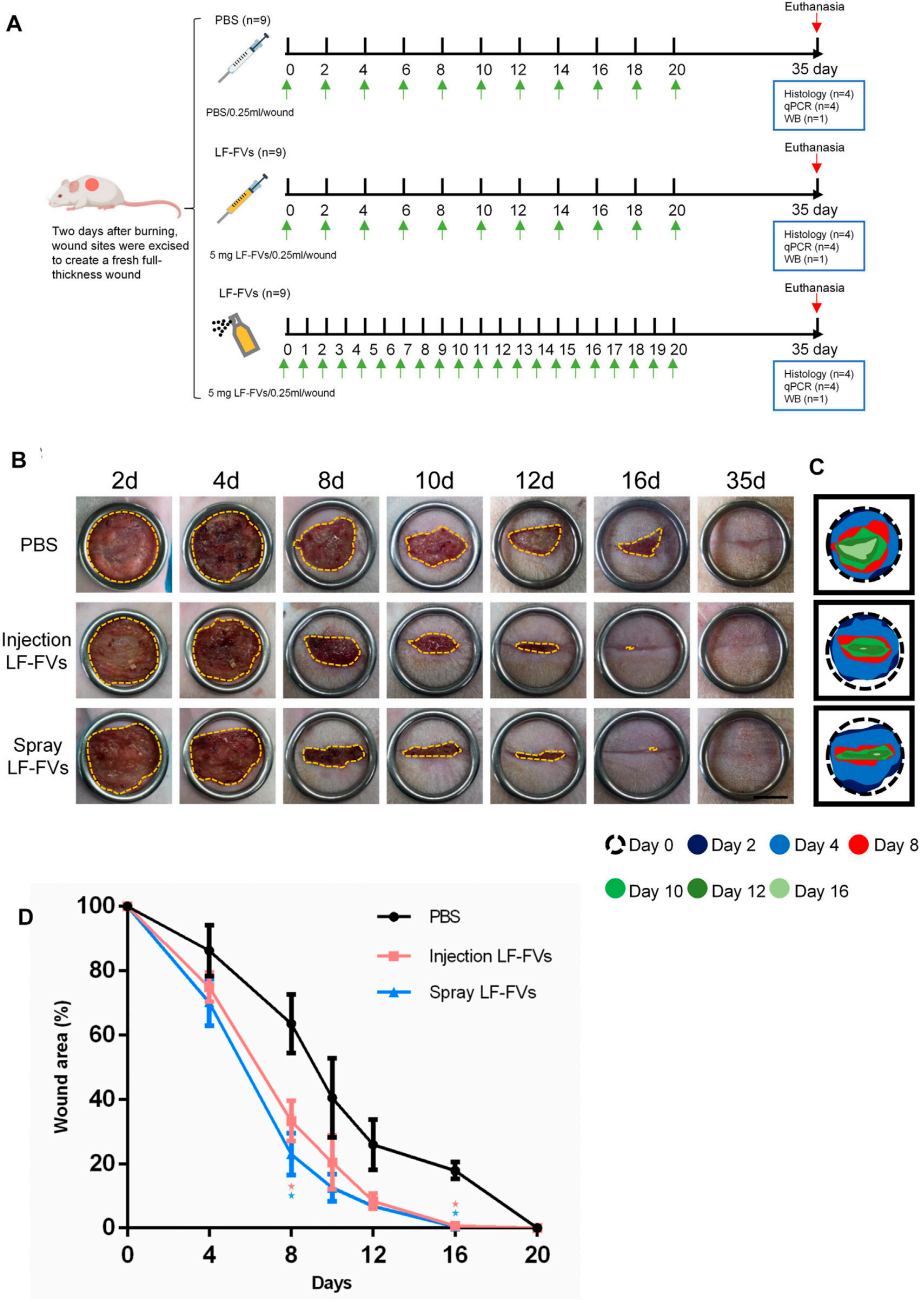
Burn wound healing was accelerated by LF-FVs. **(A)** Schematic representation of the experimental design. **(B)** Macro images of skin burn wounds at 2d, 4d, 8d,10d,12d, 16d and 35d. Scale bars = 1 cm. **(C)** Wound edge traces were established for each time point. **(D)** Quantification of the wound area. (^∗^ *p* < 0.05).

The therapeutic effects of LF-FVs through local injection or topical spray were evaluated in a rat burn model (Figure 4A). The wounds were photographed at the indicated time points (2d, 4d, 8d, 10d, 12d, 16d, and 35d) to discover the rate of wound closure (Figures 4B, C). Wound closure was faster in LF-FVs injection (31.49% ± 3.94%) and LF-FVs spray (23.63% ± 6.76%) groups compared to the PBS injection group (63.58% ± 9.13%) on day 8 (*p* < 0.05). On day 16, reepithelialization was completed in LF-FVs injection (0.85% ± 1.17%) and LF-FVs spray (0.42% ± 0.94%) groups except for the PBS injection group (17.90% ± 2.67%) (*p* < 0.05) (Figure 4D). Moreover, no significant difference was observed between the LF-FVs injection group and LF-FVs spray group at any time point during wound healing (*p* > 0.05).

### 3.4 LF-FVs improved skin appendages regeneration

**FIGURE 5:**
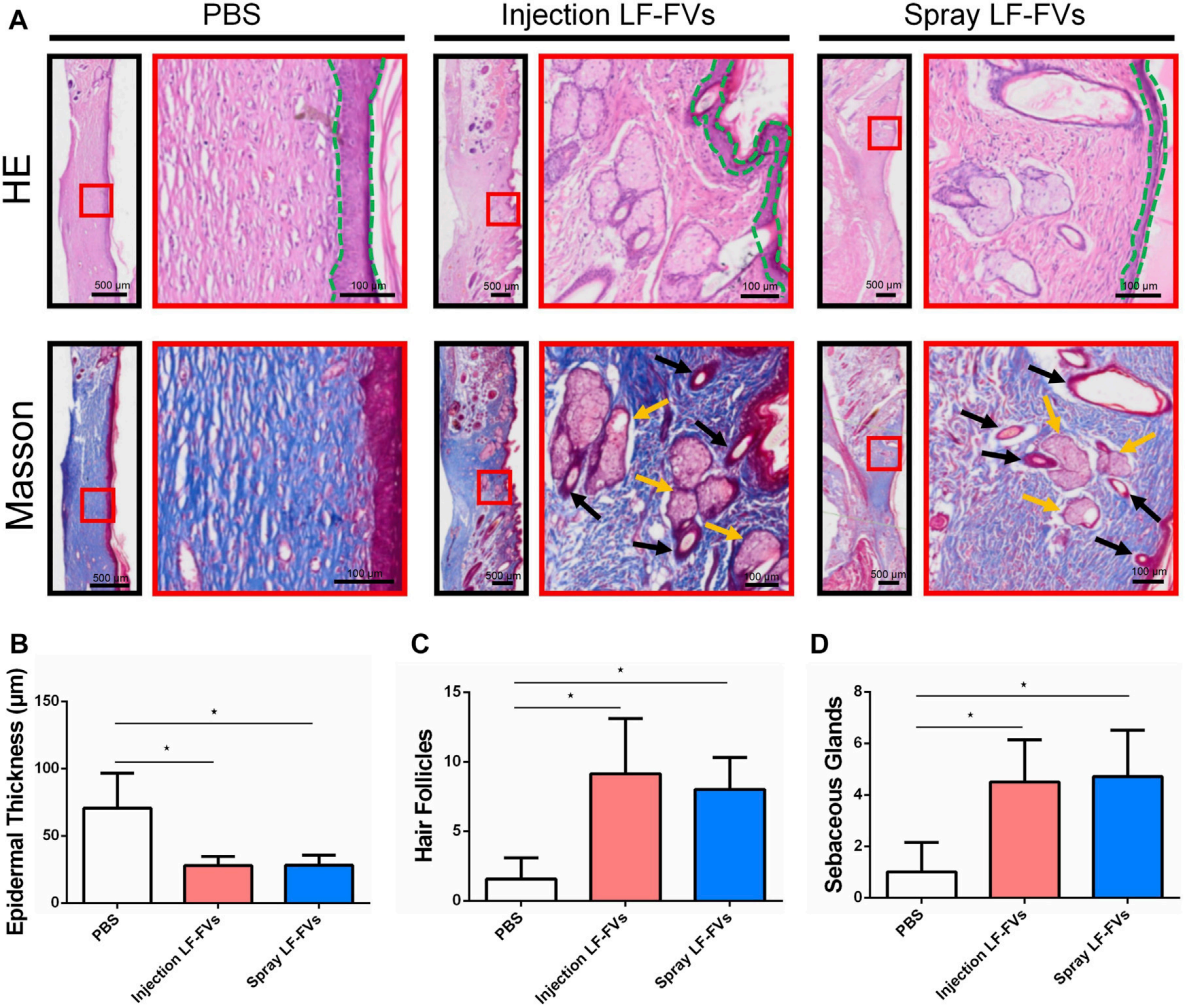
LF-FVs improved skin appendages regeneration at 35d post-treatment. **(A)** The microstructure of the healing skin was investigated by H&E and Masson staining. Green dashed lines delimit the epidermal layer in the skin tissue. Black and orange arrows mark the presence of hair follicles and sebaceous glands, respectively. Quantitative analysis of **(B)** epidermal thickness, **(C)** hair follicles and **(D)** sebaceous glands in the healing skin. (^∗^ *p* < 0.05).

At day 35 post-treatment, epithelial thickness was 70.71 ± 25.89 μm in the PBS injection group, while thinner epithelial was detected in the LF-FVs injection group (27.85 ± 6.67 μm) and LF-FVs spray group (28.11 ± 7.64 μm) (Figures 5A, B). Masson’s staining showed an increase of hair follicles in the LF-FVs injection group (5.82-fold) and LF-FVs spray group (5.09-fold) compared to PBS injection group (*p* < 0.05) (Figure 5C). The numbers of sebaceous glands in the LF-FVs injection group (4.50-fold) and LF-FVs spray group (4.71-fold) were also higher compared to PBS injection group (*p* < 0.05) (Figure 5D).

### 3.5 LF-FVs inhibited scar formation

**FIGURE 6:**
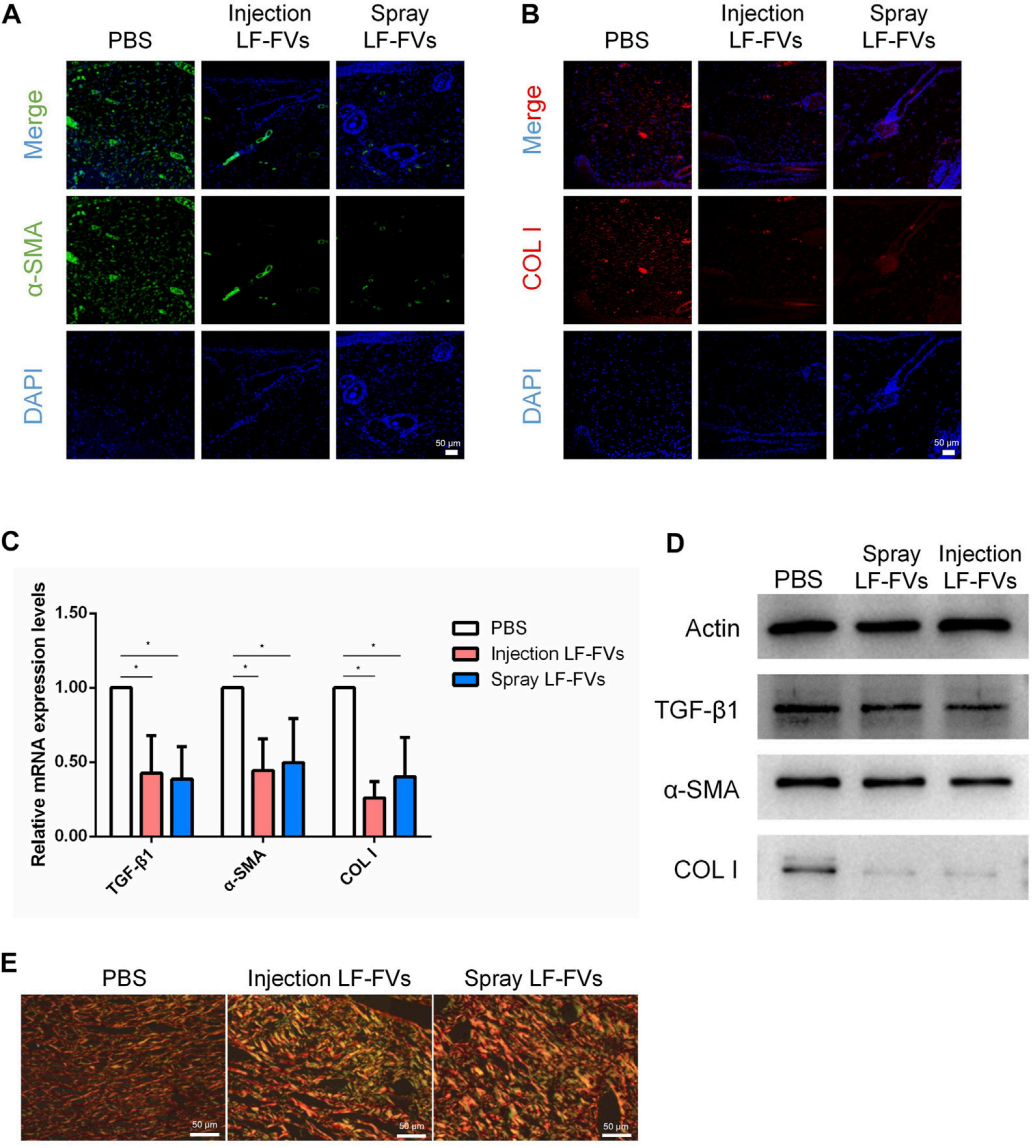
LF-FVs inhibited scar formation at 35d post-treatment. **(A, B)** Expression of α-SMA and COL I were detected using immunofluorescence staining at 35d post-treatment. Scale bars = 50 μm; magnification, ×200. **(C)** LF-FVs deceased mRNA levels of TGF-β1, α-SMA and COL I at 35d post-treatment. (^∗^ *p* < 0.05). **(D)** The expression of TGF-β1, α-SMA and COL I were detected by western blot at 35d post-treatment following LF-FVs treatment. **(E)** Skin in burn wound revealed by picrosirius red-polarization. Scale bars = 50 µm.

TGF-β1, α-SMA, and COL I are crucial scar–related molecules involved in cicatricial diseases (Desmouliere, 1993; Xiao et al., 2017; Wei et al., 2019). Immunofluorescence results implied the less presence of a-SMA and COL I (Figures 6A,B) in LF-FVs treated groups than those in the control group at day 35 post-treatment. The decreased expression of scar-related genes also was detected by real-time PCR in the LF-FVs injection group (TGF-β1, 42.50% ± 25.40%; α-SMA, 44.30% ± 21.40%; COL I, 25.90% ± 10.90%) and LF-FVs spray group (TGF-β1, 38.60% ± 21.90%; α-SMA, 49.60% ± 29.70%; COL I, 40.20% ± 26.50%) compared to the PBS injection group (*p* < 0.05), while there was no significant difference between LF-FVs injection group and LF-FVs spray group (*p* > 0.05) (Figure 6C). Western blot further confirmed the decreased levels of TGF-β1, α-SMA, and COL I in the LF-FVs treated groups (Figure 6D) Picrosirius red staining showed that COL I, which displayed the orange-red birefringence, were regularly organized in a reticular pattern in all the groups. However, bundles of COL III, which showed the green-colored birefringence, were only evident in the LF-FVs treated groups (Figure 6E).

## 4 Discussion

In recent years, studies have shown adipose tissue is not only an organ for energy storage, but also an endocrine organ (Ouchi et al., 2011). It can play an important role in the field of tissue engineering/regenerative medicine by secreting cytokines and extracellular vesicles in autocrine or paracrine manners (He et al., 2022). However, cell-based therapies have been hindered by various issues, such as cell survival, safety, immunogenicity, cell preservation, transportation and cost-efficiency for clinical applications (Poulos, 2018). Lipoaspirate fluid is a part of lipoaspirate waste and few attempts have been made to study the effects of lipoaspirate fluid. In this study, we aimed to develop a method of preparing new biological products from lipoaspirate fluid as raw material. For the first time, the present study developed a novel method to prepare a cell-free, EVs-rich product (LF-FVs) derived from human lipoaspirate fluid, which promoted the proliferation and migration of skin fibroblasts *in vitro* and accelerated burn wound healing in rats. Moreover, both early intervention with local injection or topical spray of LF-FVs improved the regeneration of burned skin and reduced scar formation.

In the past few years, extracellular vesicles have gained increasing interest from the scientific community. Studies have shown that EVs could be used in many fields of tissue repair and regenerative medicine (Nagelkerke et al., 2021; Wu et al., 2021). However, lipoaspirate fluid has not attracted extensive attention in clinical applications. There are many harvesting methods for extracellular vesicles, including sucrose gradient centrifugation; ultrafiltration; immunoaffinity magnetic bead separation; ExoQuick extraction (Tiwari et al., 2021; Wang et al., 2022). The most commonly used method is ultracentrifugation (Thery et al., 2018; Wang et al., 2019). However, ultracentrifugation results in low recovery rates, has time-consuming centrifugation steps (Coumans et al., 2017), frequently damages the EVs structure, and causes the coprecipitation of contaminants (Zheng et al., 2013). On the contrary, tangential flow filtration is operated under controlled low pressure and flow conditions (Shankaran et al., 2001), providing a suitable method to gently sieve out microparticles, avoiding sample manipulation that could lead to the formation of artificial nanoparticles during the concentration step (Paterna et al., 2022). Moreover, TFF differs from conventional dead-end filtration, as fluid flows tangentially across the surface, avoiding filter cake formation (Haraszti et al., 2018). Our study indicated that TFF was an efficient method for obtaining EVs-enriched product from lipoaspirate fluid.

LF-FVs do not contain cells, therefore can be assumed to be nonimmunogenic (Zakrzewski et al., 2014; Hu et al., 2022) and overcome challenges such as low survival rate of implanted cells or the potential risk of tumorigenicity (Ben-David and Benvenisty, 2011;; Poulos 2018). Moreover, LF-FVs do not require the use of cell cryopreservation solution which suggests that LF-FVs maybe be easily stored, transported at low temperature and serve as a potential “off-the-shelf” product for clinical use (Hu et al., 2022). In this study, xenogeneic human LF-FVs promoted burn wound healing in rats, and the results provided the possibility of future allogeneic application of LF-FVs in the clinic.

The therapeutic effect of LF-FVs on burn wounds was examined. Wound healing is a complex process usually divided into four orderly overlapping stages: clotting, inflammatory, proliferative and remodeling (Las Heras et al., 2020). Fibroblasts play an important role in scar formation and injury repair. In this study, we revealed that LF-FVs promoted the proliferation and migration of fibroblasts *in vitro* in a dose-dependent manner and LF-FVs treatment could significantly accelerate the healing of burn wounds. The potential mechanism of LF-FVs to accelerate burn wound healing may be due to the enriched EVs (Narauskaitė et al., 2021) and various active factors (Table 1) (such as adiponectin (Ryu et al., 2019)) in LF-FVs.

In this study, only the short-term effect of LF-FVs in rat third-degree burn model was observed, we plan to investigate the long-term treatment effects of LF-FVs by topical spray in a porcine burn model with optimal dosage in the future. Moreover, the application of LF-FVs could be further extended, for example, diabetic wounds, adipose tissue regeneration, skin sclerosis as well as skin rejuvenation.

## 5 Conclusion

To summarize, the present study demonstrates a novel method to prepare a cell-free and extracellular vesicle-rich liquid extract from human lipoaspirate fluid. LF-FVs, which were rich in extracellular vesicles, could promote fibroblast proliferation and migration *in vitro* and improve wound healing in a rat burn model. These findings suggest that LF-FVs might be a feasible and effective approach to inducing wound regeneration in the clinic.

## Data Availability

The original contributions presented in the study are included in the article/Supplementary Material further inquiries can be directed to the corresponding author.

## Abbreviations

CE, crude extract; COL I, collagen I; EVs, extracellular vesicles; HFF, human foreskin fibroblasts; LF-FVs, lipoaspirate fluid derived factors and extracellular vesicles; NTA, nanoparticle tracking analysis; SEC, size-exclusion chromatography; TFF, tangential flow filtration; TGF-β1, transforming growth factor-β1; α-SMA, α-smooth muscle actin.
