# Supplementary material for: Lipoaspirate fluid derived factors and extracellular vesicles accelerate wound healing in a rat burn model
Source: Front Bioeng Biotechnol. 2023 Jun 22;11:1185251. doi: 10.3389/fbioe.2023.1185251 (PMC10324973; doi:10.3389/fbioe.2023.1185251)
Supplement: Supplementary file 1 [file Table1.docx]

Supplementary Material

**Supplemental table 1**, general clinical information of donors

| **Patient** | **Gender** | **Age years** | **GLU mmol/l** | **AT depot** | **BMI** |
| --- | --- | --- | --- | --- | --- |
| 1 | female | 22 | 5.30 | waist | 19.50 |
| 2 | female | 33 | 5.27 | thigh | 23.40 |
| 3 | female | 40 | 4.70 | thigh | 20.50 |
| 4 | female | 29 | 5.02 | abdomen | 26.70 |

GLU= glucose; AT= adipose tissue; BMI=body mass index.
