# Supplementary material for: Lipoaspirate fluid derived factors and extracellular vesicles accelerate wound healing in a rat burn model
Source: Front Bioeng Biotechnol. 2023 Jun 22;11:1185251. doi: 10.3389/fbioe.2023.1185251 (PMC10324973; doi:10.3389/fbioe.2023.1185251)
Supplement: Supplementary file 2 [file Table2.docx]

Supplementary Material

**Supplemental table 2**, primers for target genes used in qPCR.

| **Gene** | **Primer sequences** |
| --- | --- |
| Rat-TGF-β1 |  |
|  | CCTGCAAGACCATCGACATG |
|  | TGTTGTACAAAGCGAGCACC |
| Rat-α-SMA |  |
|  | ATGGAGGGGAATACAGCCC |
|  | ACTACTGCCGAGCGTGAGAT |
| Rat-Col I |  |
|  | TCTGACTGGAAGAGCGGAGAG |
|  | GAGTGGGGAACACACAGGTCT |
